# Clinical commentary on ‘Cortical ischemic lesions from atrial myxoma as a mimic of disease activity in a RRMS antiCD20-treated patient’

**DOI:** 10.1177/13524585231195354

**Published:** 2023-09-15

**Authors:** Declan T Chard

**Affiliations:** NMR Research Unit, Queen Square Multiple Sclerosis Centre, Department of Neuroinflammation, UCL Queen Square Institute of Neurology, Faculty of Brain Sciences, University College London, London, UK; National Institute for Health Research (NIHR) University College London Hospitals (UCLH) Biomedical Research Centre, London, UK

In this issue of the *Multiple Sclerosis Journal*, Boccia et al.^1^ provide a timely reminder that grey matter lesions, while a clinically significant element of MS pathology, are not MS-specific. They report on a person with MS found to have an atrial myxoma after developing multiple cortical grey matter lesions (initially seen on a routine treatment monitoring magnetic resonance imaging (MRI)) while on ocrelizumab. It was with a full-body computed tomography (CT) scan, undertaken as pre-screening for autologous hematopoietic cell transplantation, that the diagnosis was made.

As the authors note, with the benefit of hindsight, there were features atypical for MS that would now give pause for further thought. The very high number of new cortical lesions and the proportion that enhanced (any enhancement is rare, and two or more lesions simultaneously would be exceedingly so^2^) would both be prompts to consider other explanations. Less useful in prospective management, but just as unexpected, more than half of the cortical lesions disappeared over about a year, whereas in MS typically ~1% are seen to do so^2^ (in contrast, most micro-embolic cortical infarcts seen on double inversion recovery MRI resolve within 6 months^3^). Interestingly, in this case, no new white matter lesions were apparent, and while intuitively this may seem unexpected, it is worth recalling that in MS the accrual of white matter and cortical grey matter lesions are not closely correlated.^2^

While an atypical presentation of a very rare tumour (the prevalence of primary cardiac tumours is estimated to be 0.02%^4^), this case highlights that we still have a lot to learn about cortical lesions in MS and other diseases. Stepping back from the case itself, the role of dedicated grey matter imaging in monitoring disease activity in MS is still evolving, and while in the authors’ practice it appears to be routine, this is not necessarily the case elsewhere: For it to become so, as with white matter lesions,^5^ we need an actionable understanding of the clinical relevance and prognostic significance of cortical lesion accrual for a person with MS.
